# Rebuilding the degenerative disc microenvironment: mesenchymal stem cells, exosomes, and bioengineered scaffolds

**DOI:** 10.3389/fbioe.2026.1802866

**Published:** 2026-05-25

**Authors:** Dong Guo, Lin Xiao, Hui Li, Ying Pan, Huan Zhang

**Affiliations:** Department of Joint Surgery, Honghui Hospital, Xi’an Jiaotong University, Xi’an, China

**Keywords:** biomaterials, exosomes, intervertebral disc degeneration, mesenchymal stem cells, regenerative medicine, tissue engineering

## Abstract

Intervertebral disc degeneration (IVDD) is a leading cause of chronic low back pain, yet current clinical interventions remain largely palliative and fail to restore disc structure and function. While mesenchymal stem cells (MSCs), exosomes, and bioengineered scaffolds have emerged as promising regenerative tools, isolated therapeutic applications often fail to overcome the highly hostile IVDD microenvironment—characterized by severe hypoxia, acidosis, and mechanical overload. This review provides a unique perspective by positioning the “cell-material-molecule” cross-integration as the central paradigm for effective disc regeneration. We critically synthesize how smart biomaterials are engineered to physically match and survive the degenerative niche, thereby providing a resilient sanctuary for MSCs. Concurrently, we highlight how engineered MSCs and their cell-free derivatives (exosomes and regulatory RNAs) synergistically dismantle inflammatory cascades and matrix breakdown. Beyond outlining preclinical and clinical advances, this review deeply analyzes persistent translational bottlenecks—such as cell source standardization, large-scale GMP exosome manufacturing, and long-term scaffold biocompatibility. Ultimately, by outlining the convergence of gene editing, responsive biomaterials, and precision delivery, we define a clear roadmap for transitioning IVDD treatment from palliative symptom management to durable, precision regenerative medicine.

## 1 Introduction

### 1.1 Global burden of IVDD and socioeconomic impact

Intervertebral disc degeneration (IVDD) is a major contributing factor to chronic low back pain, standing as one of the most prevalent and disabling musculoskeletal disorders globally and a leading cause of years lived with disability (Mohd Isa et al., 2022b; Chen et al., 2023; Jha et al., 2023). Furthermore, the rising global prevalence—accelerated by aging populations, alongside genetic, environmental, and occupational factors—translates into a profound socioeconomic burden characterized by escalating healthcare expenditures, reduced work capacity, and diminished quality of life (Samanta et al., 2023; Xia et al., 2024).

### 1.2 Pathophysiology: ECM breakdown, inflammatory microenvironment, hypoxia, biomechanics

IVDD, as the underlying pathological process, arises from a multifactorial cascade involving extracellular matrix (ECM) loss, inflammatory signaling, hypoxic stress, and altered biomechanics (Theodore, 2020; Kim et al., 2022). The intervertebral disc consists of the nucleus pulposus (NP), annulus fibrosus (AF), and cartilage endplate (CEP), each essential for mechanical integrity and nutrient exchange (Theodore, 2020; Kirnaz et al., 2022).

The pathogenesis of IVDD is driven by a complex interplay of ECM degradation, chronic inflammation, metabolic disruption, and aberrant biomechanics. Specifically, the upregulation of matrix metalloproteinases depletes proteoglycans and type II collagen, compromising NP hydration and elasticity (Theodore, 2020; Kim et al., 2022; Liang et al., 2022; Zou et al., 2023). Concurrently, an active inflammatory milieu—fueled by pro-inflammatory cytokines, macrophage infiltration, and CEP inflammation—synergizes with severe hypoxia and systemic metabolic disorders (e.g., diabetes) to accelerate NPC senescence and apoptosis (Kim et al., 2022; Song et al., 2024; Xia et al., 2024; Yang et al., 2024; Yao et al., 2024). Furthermore, biomechanical overload and endplate calcification activate mechanotransduction pathways that persistently disrupt disc homeostasis, reinforcing this comprehensive catabolic cascade (Cazzanelli and Wuertz-Kozak, 2020; Xia et al., 2024).

**FIGURE 1:**
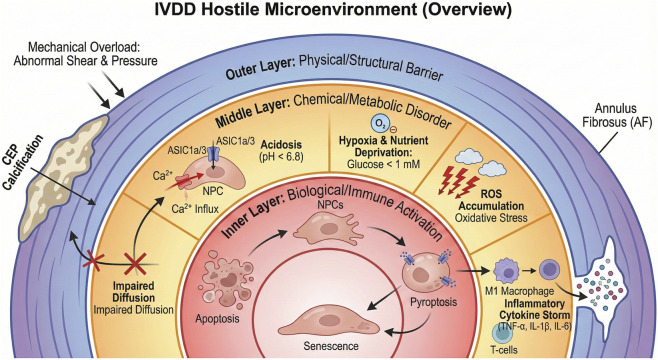
The “Trinity” hostile microenvironment and vicious cycle of IVDD. This schematic illustrates how disc homeostasis collapses across three interconnected levels. Physical stress—including mechanical overload and CEP calcification—impairs nutrient and oxygen diffusion. Chemical stress arises from chronic acidosis (pH < 6.8), severe hypoxia, glucose deprivation, and ROS accumulation, with ASIC activation driving pathological Ca^2+^ influx in nucleus pulposus cells (NPCs). Biological stress follows loss of immune privilege, allowing M1 macrophage and T-cell infiltration through AF defects and amplifying inflammation (TNF-α, IL-1β, IL-6). Together, these factors promote NPC apoptosis, pyroptosis, and senescence, ultimately leading to structural and functional disc failure.

Together, these factors converge into a “Trinity” of physical, chemical, and biological stresses, shaping a highly interconnected hostile microenvironment that drives comprehensive disc failure (Figure 1).

### 1.3 Why MSCs? Unique regenerative and immunomodulatory properties compared with other stem cells

Mesenchymal stem cells (MSCs) are distinguished from other stem cell populations by their dual regenerative and immunomodulatory functions in intervertebral disc degeneration (IVDD) (Widjaja et al., 2022). They exhibit robust multipotency, differentiating into nucleus pulposus- and chondrocyte-like cells to restore disc matrix (Ohnishi et al., 2023), while their intrinsic ability to home to injury sites and adapt to the hypoxic disc environment enhances therapeutic relevance (Ekram et al., 2021).

Beyond differentiation, MSCs exert potent paracrine activity through exosomes and small extracellular vesicles enriched with miRNAs and cytokines, which stimulate matrix anabolism and suppress catabolic and inflammatory pathways (Bhujel et al., 2022). Hypoxic preconditioning further amplifies these effects, improving regenerative efficacy (Zhou et al., 2022).

Immunomodulation is a defining feature: MSCs dampen inflammatory cascades, inhibit immune cell activation, and alleviate pain mediators, with particular benefit in inflammatory disc phenotypes (Ekram et al., 2021; Widjaja et al., 2022). Umbilical cord–derived MSCs provide additional advantages of high proliferative capacity, low immunogenicity, and ethical feasibility, supporting allogeneic use (Ekram et al., 2021). Integration and survival can be optimized by biomimetic scaffolds that replicate native extracellular matrix (Huang et al., 2023).

Overall, MSCs couple regenerative and immunomodulatory properties, offering a compelling biological approach to restore disc structure and function.

### 1.4 Knowledge gap: inconsistent clinical efficacy, limited survival, hostile microenvironment

Despite advances with mesenchymal stem cells (MSCs), exosomes, and bioengineered scaffolds, clinical translation in intervertebral disc degeneration (IVDD) remains limited. The efficacy of MSC-based therapies is inconsistent, with variable outcomes across studies due to differences in cell source, delivery route, and patient selection (Bhujel et al., 2022; Widjaja et al., 2022). Although MSCs and their exosomes can stimulate matrix synthesis and modulate inflammation, these effects are not reliably reproduced (Zhou et al., 2022).

The hostile disc microenvironment—defined by hypoxia, nutrient deprivation, acidity, and mechanical overload—severely restricts transplanted cell survival (Vadalà et al., 2019). Avascularity further impedes nutrient supply, accelerating cell death and reducing regenerative potential. Even advanced scaffolds, designed to mimic native extracellular matrix, have only partly improved retention and integration (Zhao Y. et al., 2024).

Additional hurdles include immunogenicity and potential rejection of allogeneic MSCs (Widjaja et al., 2022). Exosomes may offer safety advantages, but challenges remain with stability, targeted delivery, and sustained bioactivity (Zhou et al., 2022). Synergistic approaches combining MSCs or exosomes with hydrogels or fibrous scaffolds are under investigation, yet long-term data on regeneration, functional recovery, and durability are still sparse, and standardized protocols are lacking (Zhao Y. et al., 2024).

### 1.5 Scope of this review: mechanistic insights, preclinical and clinical advances, and emerging strategies

This review summarizes IVDD pathophysiology and highlights the regenerative potential of MSCs, exosomes, and bioengineered scaffolds. We discuss preclinical and early clinical findings and outline future directions for durable, disease-modifying therapies.

## 2 Pathophysiology of IVDD and therapeutic targets

### 2.1 Biomechanical stress and matrix breakdown

Aberrant mechanical loading is a key driver of intervertebral disc degeneration, disrupting NP and AF cell homeostasis, inducing cell death, and accelerating extracellular matrix (ECM) loss (Kibble et al., 2025; Luo et al., 2025). At the macroscopic tissue level, excessive stress directly upregulates matrix metalloproteinases and aggrecanases, which accelerates proteoglycan and collagen degradation and drives overall structural failure (Song et al., 2025a).

At the cellular and molecular level, overloading impairs autophagic flux via lysosomal dysfunction, triggering lysosome-dependent NP cell death; restoring lysosomal activity through CHMP4B or TFEB highlights lysosomal quality control as a therapeutic target (Liu et al., 2025). Mechanical and biochemical insults act together to enhance oxidative stress, with overload causing persistent ROS accumulation that links loading to apoptosis (Zhang C. et al., 2025). Transient compression may induce protective autophagy, whereas sustained overload shifts toward apoptosis and necroptosis (Khaleque et al., 2024), with ER–mitochondrial crosstalk further amplifying ROS-mediated cell death (Song et al., 2025a). Metabolic dysfunction further sensitizes disc cells to stress. Impaired glycolysis and mitochondrial activity increase vulnerability, while hyperglycemia and metabolic syndrome promote senescence-associated catabolism and apoptosis (Qin et al., 2025). Accordingly, strategies targeting oxidative stress, autophagy, and metabolism remain active therapeutic directions (Wang J. et al., 2025).

### 2.2 Inflammatory microenvironment and immune crosstalk

The inflammatory microenvironment of degenerative discs arises from the loss of immune privilege, enabling macrophage and T-cell infiltration that amplifies matrix breakdown and cell death (Ren et al., 2025). Crosstalk between these infiltrating immune cells and resident disc cells sustains cytokine production and chronic extracellular matrix (ECM) breakdown (Chen et al., 2023). Hypoxia, acidity, and cytokine signaling (e.g., NF-κB, STAT, PI3K-Akt) shape these immune phenotypes and accelerate cellular senescence (Song et al., 2025b). This immune crosstalk perpetuates chronic inflammation and ECM loss, marking the transition from immune privilege to active immune engagement in IVDD (Xu H. et al., 2023).

#### 2.2.1 Role of pro-vs. anti-inflammatory cytokines (TNF-α, IL-1β vs. IL-10, TGF-β)

Within this active immune environment, macrophage polarization is critical: M1 cells secrete pro-inflammatory mediators to drive catabolism and apoptosis, whereas M2 cells support limited tissue repair (Hou et al., 2025). Pro-inflammatory cytokines, notably TNF-α and IL-1β, are central drivers of IVDD (Zhang et al., 2022a; Dou et al., 2025). Both activate NF-κB and MAPK pathways, induce MMPs and aggrecanases, and trigger apoptosis or pyroptosis, thereby accelerating ECM loss (Wang et al., 2024; Zou et al., 2025). Single-cell and genetic studies further implicate IL-1β, IL-6, TNF, IFN-γ, and NLRP3 inflammasomes as causal mediators that amplify inflammatory cascades, reinforcing tissue damage (Zhang et al., 2022a; Zou et al., 2025). Anti-inflammatory mediators such as IL-10 and TGF-β counteract these effects (Sun K. et al., 2021; Xia et al., 2023). IL-10 suppresses TNF-α and IL-1β, while TGF-β promotes ECM anabolism and M2 polarization (Xia et al., 2023). Therapeutic strategies targeting this cytokine balance—through biologics, gene therapy, or biomaterial-based delivery—are under investigation to restore disc homeostasis (Wu B. et al., 2022).

#### 2.2.2 Macrophage polarization (M1 vs. M2) and disc resorption

Macrophage polarization between pro-inflammatory M1 and anti-inflammatory M2 phenotypes is central to IVDD (Vadalà et al., 2019). M1 infiltration, driven by hypoxia, acidity, and cytokines, promotes IL-1β, TNF-α, and MMP secretion, accelerating extracellular matrix (ECM) degradation and nucleus pulposus (NP) cell death (Li X.-C. et al., 2022; Yamamoto et al., 2022; Hou et al., 2025). In contrast, M2 macrophages secrete IL-10 and TGF-β, enhance ECM synthesis, and support repair through pathways such as OPN–CD44 and pSMAD2/3 (Yamamoto et al., 2022; Tao et al., 2025).

Crosstalk between NP cells and macrophages is bidirectional: senescent NP cells favor M1 polarization, while M1 macrophages exacerbate NP senescence (Song et al., 2025b; Xiang et al., 2025). MSC-derived exosomes carrying regulatory miRNAs and lncRNAs (e.g., miR-100-5p, *CAHM*) can reprogram M1 toward M2, reducing inflammation, apoptosis, and ECM loss *in vitro* and *in vivo* (Li W. et al., 2022; Xiang et al., 2025).

Bioengineered scaffolds and hydrogels delivering immunomodulatory agents (e.g., IL-4, EGCG) further promote M2 polarization, enhance matrix anabolism, and suppress catabolism (Cheng et al., 2022; Zhao D.-W. et al., 2024). These processes converge on signaling pathways including NF-κB, STAT, PI3K–Akt, and mTORC1, highlighting macrophage polarization as a key therapeutic target in IVDD (Zhao D.-W. et al., 2024; Chen S. et al., 2025; Xiang et al., 2025).

#### 2.2.3 Crosstalk with T cells, angiogenesis, neuroinvasion

Crosstalk between immune cells, angiogenic factors, and neural elements is central to IVDD. Single-cell analyses show that disc injury alters resident and infiltrating cell populations, upregulating T cell and angiogenic pathways and shifting MSC differentiation (Clayton et al., 2025). Loss of immune privilege permits T cells, macrophages, and neutrophils to enter *via* annulus and endplate defects, activating inflammatory cascades (Bermudez-Lekerika et al., 2022; Xu H. et al., 2023).

CD4^+^ and CD8^+^ T cells are causally linked to degeneration, driving IL-1β, TNF-α, and IL-6 release, which promote senescence and apoptosis of nucleus pulposus (NP) cells (Qin et al., 2023; Li H. et al., 2025; Ren et al., 2025; Song et al., 2025b). Hub genes such as *MAPK1* and *FOXO1* correlate with T cell and NK cell infiltration, reinforcing immune-mediated pathology (Li H. et al., 2025).

Angiogenesis, driven by hypoxia-inducible signaling and VEGF, facilitates immune entry and neural ingrowth, contributing to pain (Kim et al., 2022; Swahn et al., 2024). Thrombospondin signaling further couples fibrosis, angiogenesis, and neural invasion in NP and annulus fibrosus cells (Swahn et al., 2024).

Macrophage polarization shapes the inflammatory milieu: M1 macrophages amplify catabolism, whereas M2 macrophages support repair (Feng et al., 2023; Song et al., 2025b). Key ligand–receptor axes (SPP1–CD44, ICAM1–ITGB2) sustain inflammation, while ANXA1–FPR1 may provide counter-regulation (Shao et al., 2024). Metabolic disorders such as obesity and diabetes exacerbate these processes through chronic adipokine-driven inflammation (Francisco et al., 2022).

Breakdown of disc barriers permits neurovascular ingrowth, creating a self-sustaining cycle of degeneration and pain (Kim et al., 2022; Swahn et al., 2024). Targeted strategies—MSC transplantation, exosomes, and bioengineered scaffolds—aim to suppress immune infiltration, inhibit aberrant angiogenesis and neuroinvasion, and restore disc homeostasis (Xu H. et al., 2023; Clayton et al., 2025; Ren et al., 2025).

### 2.3 Hypoxia, acidosis, and nutrient deprivation

The avascular IVD is characterized by hypoxia, acidosis, and nutrient deprivation (Song et al., 2024). These stresses impair NP cell matrix synthesis, induce senescence and apoptosis (Kibble et al., 2025), and create a hostile microenvironment that limits regenerative approaches such as MSCs, exosomes, and scaffolds (Song et al., 2025a).

#### 2.3.1 Endplate calcification, impaired diffusion, HIF-1/2 signaling

Intervertebral disc degeneration occurs in a hostile microenvironment of hypoxia, acidosis, and nutrient deprivation, worsened by endplate calcification and impaired solute diffusion (Zehra et al., 2022). Age- and degeneration-related endplate defects reduce nutrient and oxygen transport, leading to disc cell dysfunction, matrix breakdown, inflammation, and back pain (Salman et al., 2025). Impaired diffusion shifts metabolism toward glycolysis and lactate accumulation, compromising cell viability, matrix synthesis, and inflammatory balance (Abulikemu et al., 2023). Persistent hypoxia and acidosis overwhelm HIF-1α/2α–mediated adaptation, promoting osteochondrogenic differentiation, matrix calcification, and cell death (Wu W. et al., 2022). Modulating HIF signaling, restoring endplate permeability, improving nutrition, and monitoring with diffusion MRI can support disc regeneration (Lavu et al., 2024).

#### 2.3.2 Acid-sensing ion channels, NF-κB/MAPK activation

Driven by the hostile microenvironment described above, acidic pH directly compromises NP cell function and establishes a synergistic regulatory network with inflammatory cascades. Specifically, microenvironmental acidosis upregulates acid-sensing ion channels (ASIC1a/3), triggering intracellular calcium influx and subsequent severe oxidative stress (ROS accumulation) (Zhao et al., 2021; Luo et al., 2023). Crucially, this ASIC-induced ROS burst acts as an upstream mediator that directly activates the NF-κB and MAPK signaling pathways. This cascade amplifies the release of downstream cytokines (IL-1β, TNF-α), chemokines, and matrix-degrading enzymes, creating a vicious cycle that recruits macrophages and accelerates pyroptosis and apoptosis (Burt et al., 2024). Clinical studies show NF-κB activity correlates with pain-related neuropeptides (CGRP, substance P, TRPV1) in human discs (Ahmed et al., 2019), highlighting the ASIC/ROS/NF-κB axis as a coherent target for IVDD therapy.

#### 2.3.3 Relevance to MSC survival and adaptation

The survival, proliferation, and matrix-synthesizing functions of MSCs are profoundly impaired by the hostile degenerative IVD microenvironment, particularly due to severe hypoxia, acidic pH (<6.8), nutrient deprivation, and mechanical stress (Esquijarosa Hechavarria and Richard, 2022; Zhou et al., 2022). To overcome these harsh limitations, activating intrinsic protective signaling (such as the HIF-1α/YAP pathways and STAT3 inhibition) and employing hypoxic preconditioning can synergistically enhance MSC adaptation, mitigate apoptosis and ferroptosis *via* autophagy, and augment their paracrine regenerative potential (Zhou et al., 2022; Fu S. et al., 2025). Furthermore, encapsulating these resilient MSCs within advanced bioengineered hydrogels provides a protective physical sanctuary, ultimately optimizing their long-term survival, immunomodulatory activities, and overall therapeutic efficacy (He et al., 2021; Zhou et al., 2022).

### 2.4 Genetic and epigenetic factors

Genetic and epigenetic factors drive IVDD by influencing extracellular matrix (ECM) components, inflammatory cytokines, and matrix-degrading enzymes, promoting tissue breakdown and apoptosis (Ma S. et al., 2024). Epigenetic modifications, such as DNA methylation and histone acetylation, regulate gene expression in disc cells, affecting senescence and inflammation (Liao et al., 2022). These factors also impact the efficacy of mesenchymal stem cell (MSC) therapies and exosome-based interventions, highlighting the need for personalized strategies to optimize regeneration (Wang et al., 2023).

#### 2.4.1 Risk polymorphisms

Genetic and epigenetic factors play a significant role in IVDD, with polymorphisms in collagen (*COL*) genes, growth differentiation factor 5 (*GDF5*), and the vitamin D receptor (*VDR*) gene influencing susceptibility and progression. Variants in *COL1A1*, *COL9A2*, and *COL9A3* are associated with increased risk and severity of degeneration, particularly when multiple mutations co-occur (Valtchinov et al., 2021; Bovonratwet et al., 2023). The *COL11A1* rs1676486 polymorphism has been robustly linked to heightened susceptibility, with the T allele increasing risk (Valtchinov et al., 2021). These findings highlight the multifactorial and polygenic nature of IVDD, underscoring the importance of considering gene-gene interactions and population-specific effects to improve predictive models and inform regenerative strategies.

#### 2.4.2 microRNAs (miR-640, miR-194, miR-199a, miR-532-5p), lncRNAs, ceRNA networks

Recent transcriptomic studies highlight critical roles of miRNAs, lncRNAs, and competing endogenous RNA (ceRNA) networks in IVDD (Fu Y. et al., 2025). Differentially expressed miRNAs, such as miR-640, miR-194, and miR-199a, regulate apoptosis, ECM degradation, and inflammation (Cazzanelli and Wuertz-Kozak, 2020; Li et al., 2023). For example, miR-15a-5p, upregulated in degenerative nucleus pulposus tissue, modulates cuproptosis- and ferroptosis-related genes, suggesting its potential as a biomarker (Li et al., 2023).

lncRNAs such as *H19*, *HOTAIR*, and *NEAT1* function within ceRNA networks to regulate downstream mRNA targets involved in cell death and matrix metabolism, notably by modulating JAK-STAT signaling and *COL10A1* expression (Huang et al., 2021; Wang Z. et al., 2022). Furthermore, broader ceRNA networks—including specific regulatory axes like lnc-*TMEM44-AS1*–miR-206–*HDAC4* and *MALAT1*–miR-155–*HIF1A*—govern overall disc homeostasis by influencing major intracellular pathways (e.g., PI3K-Akt, MAPK, NF-κB, and Wnt/β-catenin), thereby presenting promising targets for biomarker discovery and therapeutic intervention (Wu et al., 2023; Huang et al., 2025; Zhao W. et al., 2025).

#### 2.4.3 Exosome-mediated transfer of regulatory RNAs

Exosomes are nano-sized vesicles that mediate intercellular communication in the intervertebral disc by transferring miRNAs, lncRNAs, and circRNAs, which regulate apoptosis, senescence, inflammation, and ECM homeostasis (Li W. et al., 2022). MSC- and cartilage endplate stem cell-derived exosomes can modulate disc degeneration or regeneration; for example, miR-29b-3p from senescent CESC exosomes exacerbates oxidative stress, whereas miR-133a-3p from endplate chondrocytes and miR-221-3p from MSC exosomes mitigate degeneration by suppressing NF-κB and related pathways (Jin et al., 2025). Exosomal RNAs thus represent a promising therapeutic strategy, with ongoing efforts to optimize cargo and delivery for enhanced IVDD treatment (Gao et al., 2025).

## 3 MSC-based therapeutic strategies

### 3.1 Intradiscal injection

MSC-based intradiscal injection shows promise for IVDD, offering pain relief and functional improvement with minimal adverse events (Sayed et al., 2022). While overall clinical success rates range from 40% to 54% (Schneider et al., 2022), a critical analysis reveals that therapeutic efficacy is highly variable and heavily impacted by specific quantitative parameters: cell sources, injection dosages, and follow-up durations. For instance, the choice between autologous and allogeneic cell sources, combined with optimized dosing protocols (e.g., 10^7^ cells/disc), can dictate whether clinical improvements are transient or sustained for extended follow-up durations of up to 6 years (Lewandrowski et al., 2023). Without standardizing these variables, clinical outcomes are sometimes comparable to placebo (Sayed et al., 2022). Consequently, to improve *in vivo* retention and regenerative potential, advances in delivery methods—such as fibrous scaffolds—are increasingly utilized (Nakielski et al., 2023). Overall, while MSC therapy is a safe approach, achieving robust macroscopic disc regeneration remains a challenge that requires optimizing these core clinical parameters.

#### 3.1.1 Preclinical models: improved hydration, ECM production

Intradiscal MSC injection enhances disc hydration and extracellular matrix (ECM) production. To overcome the hostile disc microenvironment and prevent cell leakage, advanced delivery systems—such as hyaluronan-methylcellulose (HAMC) and nanostructured gelatin hydrogels—have been shown to significantly improve MSC survival, differentiation, and overall disc morphology *in vivo* (Choi et al., 2020). Furthermore, the regenerative potential of these therapies can be augmented through cell enhancement strategies, where genetically engineering MSCs (e.g., to express TGFβ1, IGF1, BMP7) or employing hypoxic preconditioning markedly boosts extracellular vesicle efficacy and matrix synthesis (Zhou et al., 2022; Kim et al., 2023).

#### 3.1.2 Clinical evidence: pain relief but limited disc height recovery

Intradiscal injection of mesenchymal stem cells (MSCs) reduces pain in IVDD but rarely restores disc height. Clinical studies report significant improvements in VAS and ODI with no serious adverse events (Lewandrowski et al., 2023; Miranda et al., 2023). However, imaging generally shows little or no recovery in disc height or volume despite symptom relief. These benefits are thought to arise mainly from immunomodulatory and anti-inflammatory effects rather than true disc regeneration. Long-term follow-up confirms durable pain reduction but persistent structural degeneration on MRI (Sayed et al., 2022).

#### 3.1.3 Risks: needle-induced injury, leakage, osteophyte formation

Needle-induced injury during intradiscal procedures can worsen disc degeneration and trigger local inflammation, though careful technique and fluoroscopic guidance reduce this risk (Sayed et al., 2022; Miranda et al., 2023). Cell leakage of MSCs or biologics may occur due to high intradiscal pressure or incomplete annular sealing, but bioengineered microscaffolds can prevent extravasation and off-target effects (Vadalà et al., 2012; Lee et al., 2023). While osteophyte formation has been observed in preclinical models, it remains a theoretical risk in humans (Bezuglov et al., 2025). Overall, intradiscal regenerative therapies show promise, but careful patient selection, injection techniques, and scaffold design are essential to ensure safety and efficacy (Lewandrowski et al., 2023; Bezuglov et al., 2025).

### 3.2 Intravenous delivery and homing

In intravenous MSC therapy for intervertebral disc regeneration, MSCs are guided to injury sites via chemotactic gradients like SDF-1 and CXCR4 (Cunha et al., 2021). Preclinical models show MSCs improve inflammation and ECM homeostasis, but their homing efficiency is limited by the disc’s low vascularity (Cunha et al., 2021). Strategies like SDF-1 hydrogels enhance MSC recruitment, but robust regeneration remains rare (Cunha et al., 2021). Despite potential, clinical translation faces challenges with cell viability and integration, with most studies focused on intradiscal injections (Lewandrowski et al., 2023). Optimization of chemotactic signaling and exosome engineering is essential for improving outcomes (Ohnishi et al., 2023).

#### 3.2.1 Immunomodulation over structural regeneration

Intravenous MSC delivery primarily exploits their immunomodulatory properties and homing capabilities (*via* the SDF-1/CXCR4 axis) to navigate to injury sites (Vadalà et al., 2019). Once engrafted, MSCs actively counteract the catabolic cytokine cascades (as detailed in Section 2.2.1) by secreting robust levels of anti-inflammatory mediators like IL-10 and TGF-β, thereby reprogramming the hostile niche into a microenvironment favorable for repair (Vadalà et al., 2019; Zhao Y. et al., 2024). They also differentiate into disc-like cells and synthesize ECM components such as aggrecan and type II collagen, essential for restoring disc function (Vadalà et al., 2019; Nakielski et al., 2023). In large animal models, intravenous MSCs have shown improved disc height and matrix deposition, with bioengineered scaffolds enhancing MSC retention and viability (Nakielski et al., 2023). MSCs also release exosomes that stimulate cell proliferation, inhibit apoptosis, and promote matrix regeneration, further supporting their therapeutic efficacy in disc repair (Zhao Y. et al., 2024). This strategy, combining MSCs, homing, and scaffolds, offers a promising approach for treating IVDD (Nakielski et al., 2023).

#### 3.2.2 Chemotactic factors (SDF-1/CXCR4, CCL5)

Chemotactic factors, particularly SDF-1/CXCR4 and CCL5, play key roles in recruiting and activating stem/progenitor cells during IVDD. The SDF-1/CXCR4 axis is upregulated during IVDD, enhancing migration and proliferation of MSCs and nucleus pulposus-derived stem cells (NPSCs) in response to proinflammatory signals (Zhang et al., 2018; Ying et al., 2019). Blocking CXCR4 with antagonists like AMD3100 confirms the specificity of this pathway (He et al., 2018; Zhang et al., 2018). Hyaluronan-based hydrogels delivering SDF-1 improve MSC recruitment and migration, while CXCR4-overexpressing MSCs enhance matrix protein expression and disc height preservation (Pereira et al., 2014; Leite Pereira et al., 2018). Additionally, CCL5 acts as a potent chemoattractant for MSCs and enhances ECM synthesis in *ex vivo* models (Pattappa et al., 2014; Frapin et al., 2020). These chemotactic factors also modulate inflammation and promote matrix regeneration, supporting their therapeutic potential for disc degeneration, though optimal delivery strategies are still needed (Ekram et al., 2021).

#### 3.2.3 Engineering strategies to enhance homing efficiency

Engineering strategies to improve MSC homing to the degenerative disc focus on optimizing cell recruitment, survival, and integration. Chemoattractant-based systems like hyaluronan/SDF-1 hydrogels and fibrous microscaffolds have shown promise in increasing local cell numbers and preventing leakage (Cunha et al., 2021; Nakielski et al., 2023). MSC homing is enhanced by chemokine gradients, microenvironmental cues, and antioxidant encapsulation, which protect against oxidative stress and improve cell survival (Beeravolu et al., 2018; Fang et al., 2024). Genetic engineering, such as CRISPR/Cas9 for cytokine delivery, further enhances regenerative effects (Kim et al., 2023). MSC-derived exosomes and scaffolds improve cell proliferation and tissue regeneration, while functionalized bioscaffolds address the harsh IVD microenvironment (Beeravolu et al., 2018; Sun Y. et al., 2021). These strategies collectively aim to optimize MSC homing and enhance regenerative potential for disc degeneration.

### 3.3 Genetic modification and preconditioning of MSCs

Current strategies to enhance MSCs for intervertebral disc regeneration include genetic modification and preconditioning. CRISPR/Cas9-mediated genetic editing of cytokines like TGFβ1, IGF1, and BMP7 improves disc cell regeneration, ECM restoration, and inflammation suppression (Kim et al., 2023). Co-culturing MSCs with degenerative disc cells also boosts anti-inflammatory mediators like IL-10 (Dalton et al., 2025). Preconditioning MSCs with stressors like hypoxia or antioxidants increases their survival and regenerative efficacy, with hypoxic preconditioning enhancing small extracellular vesicle (sEV) production and pharmacologic preconditioning protecting MSCs from oxidative stress (Hu et al., 2023; Fang et al., 2024). Both approaches improve MSC survival, differentiation, and regenerative potential in disc degeneration (Hu et al., 2023).

#### 3.3.1 Hypoxic preconditioning, inflammatory preconditioning

To counter the severe hypoxic and inflammatory stress intrinsic to the degenerative IVD (detailed in Sections 2.2, 2.3), preconditioning strategies are actively employed to enhance MSC resilience prior to transplantation. Specifically, hypoxic preconditioning (culturing MSCs in 1%–5% O_2_) improves cellular survival, proliferation, and chondrogenic differentiation by pre-adapting them to the hostile disc niche (Yang et al., 2022; Khaswaneh et al., 2025). Furthermore, inflammatory preconditioning with cytokines (e.g., TNF-α or IL-1β) improves MSC homing and amplifies their immunomodulatory paracrine activity (McKinney et al., 2022). Both strategies increase MSC resilience to oxidative stress and nutrient deprivation, conditions typical of degenerative discs, and are often combined with genetic modification techniques like CRISPR/Cas9 to further enhance their regenerative potential (Moeinabadi-Bidgoli et al., 2023). These approaches have shown significant benefits in preclinical models, optimizing MSC function for disc regeneration (Sun et al., 2023).

#### 3.3.2 Gene editing (CRISPR/Cas, PI3K/Akt pathway enhancement)

Gene editing technologies, particularly the CRISPR/Cas systems, have revolutionized MSC therapies by enabling precise manipulation of both regenerative and inflammatory pathways. By upregulating key anabolic genes (e.g., *TGFB1*, *IGF1*, *BMP7*, *ACAN*, and *ZNF865*) and activating pro-survival signaling like the PI3K/Akt pathway, CRISPR/Cas9 modifications significantly enhance MSC survival, robust ECM production, and overall tissue biomechanics under degenerative conditions (Matta et al., 2021; Kim et al., 2023; Levis et al., 2023; Levis et al., 2025). Beyond traditional gene editing, advanced CRISPRi-mediated epigenome editing has emerged as a powerful tool to directly silence inflammatory cascades, effectively reducing pain and structural degeneration *in vivo* (Stover et al., 2024).

#### 3.3.3 Improved survival, differentiation, and paracrine activity

Mesenchymal stem cells (MSCs) are promising for intervertebral disc degeneration (IVDD) due to their ability to survive, differentiate, and modulate inflammation within the disc microenvironment (Fu S. et al., 2025). Encapsulation of MSCs in matrix hydrogels reduces oxidative stress-induced ferroptosis in annulus fibrosus cells, improving viability and slowing IVDD progression (Fu S. et al., 2025). MSC-derived exosomes, particularly under hypoxic conditions, deliver microRNAs like miR-17-5p to nucleus pulposus cells, promoting matrix synthesis *via* the TLR4/PI3K/AKT pathway (Fu S. et al., 2025). Exosomes also modulate immune responses by regulating macrophage polarization and reducing inflammation, which supports tissue regeneration and ECM preservation (Li W. et al., 2022). MSC therapies enhance regeneration by increasing the Tie2-positive progenitor cell population and improving disc cell function through paracrine signaling (Wangler et al., 2019). Bioengineered scaffolds, such as GelMA hydrogels, further support MSC viability and matrix deposition under mechanical loading (Li X. et al., 2025).

## 4 MSC-derived exosomes: cell-free therapeutics

### 4.1 Intracellular regulation of nucleus pulposus cells: apoptosis, autophagy, and redox balance

MSC-derived exosomes act as cell-free therapeutics in IVDD primarily by preserving nucleus pulposus cell (NPC) viability against the hostile microenvironment. Mechanistically, specific exosomal cargos (e.g., miR-21, miR-17-5p) strongly activate the pro-survival PI3K/Akt axis, while others (such as miR-125-5p, miR-217, and miR-100-5p) enhance protective autophagic flux *via* mTORC1 and FOXO3 signaling, collectively preventing NPC degeneration (Cheng et al., 2018; Chen and Jiang, 2022; Hao et al., 2022; Xiao et al., 2022). Concurrently, exosomes mitigate severe oxidative stress by scavenging reactive oxygen species (ROS) and stabilizing the Nrf2 antioxidant defense pathway (e.g., *via* m6A demethylation or delivering MnSOD), effects that can be further amplified by oxidative preconditioning (Yao et al., 2019; Li et al., 2021; Rahimi et al., 2021; Kang et al., 2022). Furthermore, a diverse array of exosomal RNAs (including miR-532-5p and miR-142-3p) synergistically suppress intracellular apoptotic cascades and temper endoplasmic reticulum (ER) stress to stabilize overall matrix homeostasis (Liao et al., 2019; Zhu G. et al., 2020; Zhu L. et al., 2020).

### 4.2 Extracellular immunomodulation: macrophage reprogramming and inflammatory cascades

**FIGURE 2:**
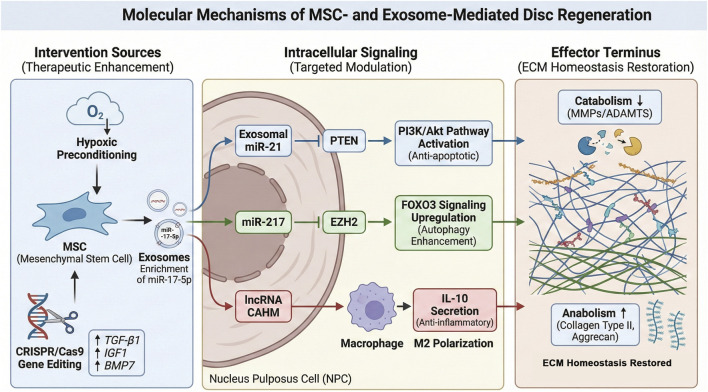
Molecular mechanisms of engineered MSC-exosome interventions in disc regeneration. This schematic illustrates how engineered MSC-derived exosomes modulate nucleus pulposus cells (NPCs). Hypoxic preconditioning enriches exosomal miR-17-5p, while CRISPR/Cas9 editing enhances regenerative factors such as TGF-β1, IGF1, and BMP7. Key targets include miR-21–mediated PTEN inhibition to activate PI3K/Akt signaling, miR-217 suppression of EZH2 to promote FOXO3-dependent autophagy, and lncRNA *CAHM*–induced M2 macrophage polarization to reduce inflammation. Together, these pathways restore ECM homeostasis by suppressing MMPs/ADAMTS and enhancing type II collagen and aggrecan synthesis, shifting the disc toward a regenerative phenotype.

Beyond intracellular protection, MSC-derived exosomes profoundly alter the extracellular inflammatory milieu, primarily by reprogramming macrophage polarization. Addressing the pivotal role of macrophages in IVDD pathology (as extensively discussed in Section 2.2.2), exosomal cargos such as lncRNA *CAHM* directly shift macrophages from a pro-inflammatory M1 toward a reparative M2 phenotype, effectively halting ECM degradation (Li W. et al., 2022; Xiang et al., 2025). Moreover, exosomes deliver a payload of bioactive miRNAs (e.g., miR-148a-3p, miR-152-3p, and miR-410) that systematically dismantle inflammatory cascades by inhibiting the NF-κB and MAPK pathways and suppressing NLRP3 inflammasome-mediated pyroptosis (Zhang et al., 2020; Zhu L. et al., 2020; Li X. et al., 2025). By systematically coordinating intracellular pro-survival signaling (e.g., PI3K/Akt, autophagy) and extracellular immunomodulation (resolving cytokine storms), these engineered cell-free therapeutics establish a highly permissive environment for robust matrix homeostasis and disc regeneration (Xia et al., 2019; Xu G. et al., 2023) (Figure 2).

Beyond these qualitative mechanisms, the quantitative efficacy of MSC-derived exosomes is striking. *In vitro*, engineered exosomes targeting specific lncRNAs or miRNAs have been shown to quantitatively suppress the expression of pro-inflammatory cytokines (e.g., IL-1β, TNF-α) by over 50%–70% relative to untreated degenerative models (Zhu L. et al., 2020; Li X. et al., 2025). Translating *in vivo*, robust preclinical rodent studies demonstrate that these cell-free interventions can restore the macroscopic Disc Height Index (DHI) by approximately 60%–80% compared to healthy controls, significantly outperforming unguided, basic MSC injections in preventing structural collapse (Wei et al., 2026). To further maximize this therapeutic efficacy, artificial intelligence (AI) and machine learning algorithms are increasingly being integrated into exosome engineering. Rather than relying on traditional trial-and-error, AI-driven predictive modeling can analyze vast transcriptomic datasets to identify the most potent miRNA/lncRNA combinations and simulate optimal exosome loading schemes. This computational approach streamlines the design of customized, high-yield cell-free therapeutics, establishing a critical technological bridge toward precision medicine (Hong, 2022; Trapana et al., 2024).

### 4.3 Pharmacokinetic and biodistribution limitations in the IVD microenvironment

While the intracellular regulatory mechanisms of MSC-derived exosomes are increasingly well-documented, their clinical translation as a definitive “cell-free therapy” is fundamentally constrained by a lack of rigorous pharmacokinetic data. Treating exosomes as pharmacological agents requires a comprehensive understanding of their absorption, distribution, metabolism, and excretion (ADME). However, current preclinical studies rarely quantify these parameters (Jin et al., 2025; Wei et al., 2026).

Following intradiscal injection, native exosomes exhibit a notoriously short half-life and are subject to rapid *in vivo* clearance (Lu et al., 2021). Furthermore, their cellular uptake efficiency is profoundly challenged by the unique physical environment of the intervertebral disc. The dense, avascular extracellular matrix acts as a physical barrier, while the high osmotic pressure and dynamic cyclical loading of the spine actively squeeze these nanovesicles out of the disc space, driving off-target distribution into the systemic circulation (Vadalà et al., 2012; Nakielski et al., 2023).

Consequently, systematically mapping the biodistribution and defining the precise degradation kinetics of exosomes under physiological mechanical stress remains a critical blind spot in the field. Addressing these pharmacokinetic limitations is just as important as deciphering their molecular cargo, underscoring the necessity of developing sustained-release biomaterial carriers (as discussed in Section 5) to maintain localized therapeutic concentrations (Tang et al., 2025; Zhang J. et al., 2025).

## 5 MSCs and biomaterial-based disc regeneration

### 5.1 Injectable hydrogels as cell carriers

Injectable hydrogels are designed to mimic the native nucleus pulposus (NP) matrix, creating a supportive niche that improves MSC survival, differentiation, and extracellular matrix regeneration (Chen Y. et al., 2025). Incorporation of bioactive molecules such as antioxidants or growth factors further protects MSCs from oxidative stress and apoptosis, enhancing their reparative capacity in degenerative disc models (Fang et al., 2024). In addition to cytocompatibility, these hydrogels provide mechanical stability and reduce cell leakage under disc pressure, supporting structural repair *in vivo* (Cherif et al., 2024).

#### 5.1.1 Natural and synthetic hydrogel matrices

Injectable hydrogels provide a supportive 3D niche for MSC survival, yet their microenvironmental adaptability heavily depends on matching specific physicochemical properties to the degenerative niche (Nishiguchi et al., 2024). Natural hydrogels (e.g., collagen, gelatin, and hyaluronic acid) enhance MSC adhesion by mimicking the native extracellular matrix (Edwards et al., 2024). However, they often exhibit rapid, uncontrolled degradation rates and compromised mechanical strength in the severe acidic (pH < 6.8) and MMP-rich degenerative microenvironment. Conversely, synthetic matrices offer tunable stiffness and maintain prolonged mechanical stability under hypoxic and acidic conditions (Martin et al., 2023), but may lack intrinsic bioactive motifs. To resolve these adaptability deficiencies, hybrid composites and advanced crosslinking techniques integrate these advantages to maximize injectability and pro-regenerative effects (Yao et al., 2022). Crucially, extensive *in vivo* preclinical evaluations—ranging from rodent models to large animals (sheep and goats)—consistently confirm that these advanced matrices significantly improve MSC viability, prevent cell leakage under high disc pressure, and effectively stabilize disc height and biomechanics (Schmitt et al., 2021; Tang et al., 2025; Zhang Y. et al., 2025).

#### 5.1.2 Functionalized hydrogels for controlled release

Injectable hydrogels provide a 3D matrix for MSC survival and differentiation while serving as delivery vehicles for growth factors (Cheon et al., 2024). Their composition, crosslinking, and integrin-binding motifs (e.g., RGD) influence mechanics, degradation, and cell–matrix interactions (Dvořáková et al., 2021). Controlled-release systems, including supramolecular, peptide-based, and stimuli-responsive hydrogels, enable sustained or on-demand delivery of bioactive molecules like TGF-β1 and VEGF-A, enhancing chondrogenesis, MSC retention, and matrix regeneration (Li Q. et al., 2022).

### 5.2 Tissue-engineered scaffolds

Tissue-engineered scaffolds mimic native disc architecture and provide a bioactive niche that supports MSC survival and differentiation (Nakielski et al., 2023). Hydrogel and composite based systems, such as chitosan or polycaprolactone–hydrogel constructs, enhance matrix synthesis and address challenges of hypoxia and mechanical stress in degenerated discs (Zhao Y. et al., 2024).

#### 5.2.1 Decellularized ECM, genipin-crosslinked hydrogels

Decellularized extracellular matrix (dECM) scaffolds preserve native disc architecture and bioactivity, guiding MSC differentiation toward NP- or AF-like phenotypes *via* TGF-β signaling (Fiordalisi et al., 2021). Injectable dECM hydrogels enable minimally invasive delivery, with genipin crosslinking improving stability, elasticity, and cell viability while promoting ECM synthesis and disc regeneration *in vivo* (Xu et al., 2019). Composite systems, such as genipin-crosslinked fibrin or alginate microbeads, further enhance mechanical support, reduce apoptosis, and sustain matrix production in long-term models (Panebianco et al., 2022). Recent designs combining dECM with PEG or silk offer tunable degradation and bioactive release, though challenges remain in standardization and clinical translation (Schmitz et al., 2023).

#### 5.2.2 Self-assembling peptides, nanofiber scaffolds, microfluidic microspheres

Advanced nanoscale and microfluidic platforms offer unprecedented control over scaffold architecture and bioactive delivery. For instance, self-assembling peptides (SAPs) and their glycosaminoglycan (GAG) hybrids form injectable, nanofibrous hydrogels that not only restore macroscopic disc biomechanics but can also be functionalized to deliver growth factors (e.g., BMP7, bFGF) to promote robust NP/AF cell proliferation and ECM synthesis (Gelain et al., 2020; Tu et al., 2023). Similarly, electrospun nanofiber scaffolds engineered with angle-ply or core–shell architectures precisely replicate the native AF microenvironment while providing sustained release of anti-inflammatory cues (Li C. et al., 2022). Furthermore, injectable microfluidic microspheres (such as HAMA or GelMA-CS) provide highly controlled, compartmentalized delivery of MSCs and drugs, synergistically modulating oxidative stress and promoting NP-like matrix regeneration (Ma T. et al., 2024).

### 5.3 Bioengineered artificial discs

Bioengineered artificial discs, including whole-disc constructs like engineered disc-like angle-ply structures (eDAPS), combine biomaterials, scaffold design, and progenitor cells to replicate native disc structure and function (Frehner et al., 2025; Yang et al., 2025). However, the evaluation of these constructs must extend beyond simple static load-bearing capacity to a rigorous mechanobiology perspective (Cazzanelli and Wuertz-Kozak, 2020). To avoid the deposition of disorganized, scar-like fibrotic tissue, artificial discs must replicate the dynamic mechanical behaviors of the native IVD, including viscoelasticity, nonlinearity, anisotropy, and frequency-dependent stress relaxation. Crucially, the physical architecture of the scaffold dictates how physiological mechanical signals are transmitted to the loaded MSCs. By engineering anisotropic microstructures for the annulus fibrosus and viscoelastic, energy-dissipating cores for the nucleus pulposus, advanced scaffolds can accurately transduce dynamic spinal loads into intracellular mechanotransduction signals (e.g., *via* focal adhesion kinase and YAP/TAZ pathways) (Mordechai et al., 2023; Wang et al., 2023). This precise mechanical signaling at the cell-scaffold interface is essential to direct MSCs toward synthesizing a correct, native-like extracellular matrix with the proper microstructural hierarchy, rather than driving an aberrant repair phenotype (Mordechai et al., 2023; Wang Z. et al., 2025).

#### 5.3.1 Silk fibroin, PCL-HA composites, 3D bioprinting

Silk fibroin, polycaprolactone-hydroxyapatite (PCL-HA) composites, and 3D bioprinting represent key platforms for bioengineered artificial discs. Silk fibroin scaffolds support MSC adhesion, proliferation, and ECM synthesis with good *in vivo* integration and minimal inflammation (Mohd Isa et al., 2022a). PCL-HA composites, fabricated by electrospinning or 3D printing, provide tunable mechanics and osteoconductivity, enhancing MSC viability and matrix deposition, with animal studies showing preserved disc height and substantial tissue repair (Zhu et al., 2021). Crucially, 3D bioprinting precisely addresses the microenvironmental conflict between nutrient diffusion and biomechanical loading; it enables targeted spatial tuning of scaffold porosity to facilitate oxygen transport in the avascular niche, while simultaneously matching the mechanical stiffness to withstand cyclical physiological loads without structural collapse (Nakielski et al., 2023).

Recently, the development of these advanced composite scaffolds has been significantly accelerated by the integration of AI and machine learning (Kennedy et al., 2025). AI algorithms are now utilized for high-throughput screening of hydrogel formulations, optimizing 3D printability, and predicting long-term degradation kinetics (Silva Robazzi et al., 2025). Furthermore, computational modeling can simulate how specific 3D bioprinted topologies and localized mechanical gradients will dictate MSC differentiation trajectories *in vivo* (Spagnuolo et al., 2026). This AI-driven material optimization ensures that scaffolds actively guide targeted tissue remodeling rather than merely providing passive structural support (Hong, 2022; Mohd Isa et al., 2022a).

#### 5.3.2 Composite scaffolds mimicking annulus-nucleus architecture

**FIGURE 3:**
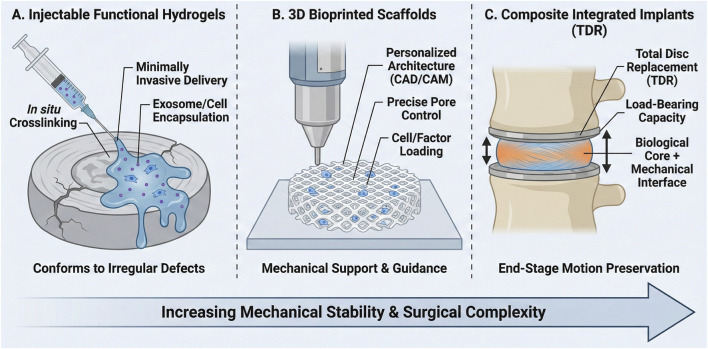
Bioengineering strategies for IVD regeneration. **(A)** Injectable hydrogels provide a protective niche, improving MSC survival, retention, and sustained release of factors such as TGF-β and BMP-2. **(B)** 3D scaffolds and advanced 3D bioprinting enable precise spatial control of cells and biomaterials, providing mechanical support, guiding MSC differentiation into NP- or AF-like cells, and promoting type II collagen and aggrecan synthesis. **(C)** Composite integrated implants and artificial discs, such as silk fibroin- or PCL-HA-based systems, replicate AF–NP architecture, restore disc height and hydration, and support functional motion preservation.

Alongside injectable hydrogels and 3D bioprinting technologies summarized in Figure 3, composite scaffolds that recapitulate the holistic annulus fibrosus (AF)–nucleus pulposus (NP) architecture are central to comprehensive IVD regeneration. Advances in 3D printing and electrospinning have enabled region-specific constructs, such as PLA frameworks for AF with cell-laden hydrogels for NP, achieving native-like mechanics and porosity (Zhu et al., 2021). Angle-ply PCL scaffolds and silk-reinforced composites reproduce AF anisotropy and support cell adhesion *in vivo*, while biphasic collagen-GAG systems mimic disc biomechanics and dynamic recovery (Mordechai et al., 2023).

Injectable composites, including genipin-crosslinked fibrin with alginate microbeads, balance stability and viability, promoting AF repair and NP matrix synthesis (Dai et al., 2025). Electrospun nanofibrous scaffolds with chitosan or chondroitin sulfate enhance bioactivity under disc pressure, and esterase-responsive NP-like hydrogels provide antioxidant protection and preserve disc height *in vivo* (Nakielski et al., 2023).

Engineered discs combining porous PCL foam for AF and HA hydrogels for NP maintain structure and function when seeded with MSCs, while negative Poisson’s ratio scaffolds with drug delivery mitigate inflammation and NP protrusion (Sha et al., 2025). Integrated AF-NP scaffolds with oriented PCL fibers and alginate cores further support region-specific colonization and ECM formation (Du et al., 2019). Hybrid nanoscaffolds with enzyme-mimetic nanosheets regulate inflammation and oxidative stress, restoring stiffness and disc function in preclinical models (Yang et al., 2023).

#### 5.3.3 Current barriers: biomechanics, integration, large-animal validation

Bioengineered artificial discs face key translational barriers in biomechanics, integration, and large-animal validation. Achieving physiological load-bearing remains difficult; PCL–hydrogel composites restore disc height short-term but show proteoglycan loss and incomplete remodeling (Kim et al., 2020). Integration with host tissue is limited by poor cell infiltration and high-pressure leakage, despite improvements with fibrous micro-scaffolds (Nakielski et al., 2023). The hostile disc microenvironment (hypoxia, acidity, hyperosmolarity) further compromises MSC survival and ECM synthesis, underscoring the need for biomimetic scaffolds (Vadalà et al., 2019; Zhao Y. et al., 2024). Large-animal studies in sheep and goats are essential to test long-term stability and immunogenicity, yet current data show incomplete matrix retention and integration despite disc height recovery (Li X. et al., 2025). Addressing these barriers will require iterative biomaterial design, cell engineering, and rigorous large-animal validation before clinical translation (Huang et al., 2023). To substantiate the biological efficacy of these interventions, Table 1 provides a condensed quantitative analysis of representative studies. By highlighting precise models, dosages, and statistical outcomes, this synthesis underscores the current therapeutic potential and translational gaps in IVD regeneration.

**TABLE 1:** Quantitative synthesis of representative regenerative strategies for IVDD.

Intervention	Model and endpoint	Parameters and dosage	Quantitative outcomes	Key limitations	References
Intradiscal MSCs	Human (n = 45); 12–72 months	BM/UC-MSCs; ∼10^7 cells/disc	VAS: 7.8 to 2.4 (p < 0.001); improved ODI	Minimal DHI reversal on MRI	Sayed et al. (2022), Schneider et al. (2022), Lewandrowski et al. (2023)
MSC-Exosomes	Rat tail; 8–12 weeks	Engineered EVs; 50–100 μg	60%–80% DHI recovery (p < 0.05); >50% cytokine drop	Rapid *in vivo* clearance	Zhang et al. (2020), Li W. et al. (2022), Li X. et al. (2025); Wei et al. (2026)
Hydrogel + MSCs	Rat tail; 12 weeks	WJ-MSCs in HAMC	>80% cell retention (2 weeks); preserved disc morphology	Poor endplate integration	Choi et al. (2020), Schmitt et al. (2021), Tang et al. (2025)
3D Scaffolds	Porcine/Rodent; 6–12 weeks	BM-MSCs in Scaffolds	∼85% mechanical restoration; >40% cell retention	Complex manufacturing	Kim et al. (2020), Mordechai et al. (2023), Nakielski et al. (2023)

## 6 Translational challenges and future perspectives

A critical synthesis of current literature reveals a profound disparity in the relative strength of evidence across translational stages. Human clinical trials predominantly reflect immunomodulatory symptom management (e.g., pain relief) rather than true macroscopic disc height recovery (Sayed et al., 2022; Lewandrowski et al., 2023; Miranda et al., 2023). To navigate this unresolved controversy, it is essential to clearly distinguish between biological “repair” and true “regeneration.” Repair typically involves the deposition of disorganized, scar-like fibrocartilage (often rich in Type I collagen) that may temporarily increase matrix volume on MRI but fails to restore native biomechanics. In contrast, functional regeneration demands the restoration of the disc’s native hierarchical architecture (anisotropic AF and a gelatinous, Type II collagen/aggrecan-rich NP) (Binch et al., 2021). Current evidence suggests that clinical pain relief primarily stems from MSC/exosome-mediated anti-inflammatory and immunomodulatory effects, while observed matrix increases on imaging often represent limited repair rather than functional regeneration (Binch et al., 2021; Zhang et al., 2022b). This critical gap between limited biological repair and true functional regeneration underscores the fundamental challenge of translating therapies into the highly hostile, biomechanically demanding human disc microenvironment (Binch et al., 2021). Current evidence strongly suggests that the clinical pain relief observed with MSC and exosome therapies primarily stems from their potent anti-inflammatory and immunomodulatory effects (e.g., macrophage reprogramming), rather than structural rebuilding. Furthermore, while composite scaffolds and artificial discs frequently demonstrate “matrix increases” on preclinical imaging, histological evidence often reveals that this is limited fibrotic repair rather than true functional regeneration (Zhang et al., 2022b). This critical gap between limited biological repair and true functional regeneration underscores the fundamental challenge of translating therapies into the highly hostile, biomechanically demanding human disc microenvironment.

### 6.1 Challenges in standardization

Cell sources, dosing, and delivery methods vary widely across studies, hindering comparability and reproducibility (Kabat et al., 2020). Standardization remains a major barrier, as the functional potency of MSCs is profoundly dictated by tissue origin, donor age, and passage number (Calcat-I-Cervera et al., 2023). Advanced donor age and extended passage numbers (e.g., beyond P5) quantitatively reduce MSC proliferation—often by 2- to 3-fold—and impair chondrogenic capacity (Bagge et al., 2022). Mechanistically, this senescence is driven by the overactivation of the Wnt/β-catenin pathway and the concurrent suppression of TGF-β/Smad signaling, which diminishes the secretion of anabolic factors (Zhang et al., 2022b; Huang et al., 2023). Clinically, these cell sources face distinct regulatory landscapes. Autologous BM-MSCs and AD-MSCs dominate early trials as they often meet ‘minimal manipulation’ criteria (Binch et al., 2021; Ma C.-Y. et al., 2024). Conversely, while UC-MSCs offer scalable banking potential, they require rigorous IND approval to guarantee GMP compliance (Ma C.-Y. et al., 2024). Exosome standardization similarly requires transitioning to precise quantitative metrics. Different isolation platforms dramatically affect the therapeutic product; for example, size-exclusion chromatography (SEC) quantitatively improves the particle-to-protein purity ratio (often by >-fold) compared to conventional ultracentrifugation (UC), ensuring a narrower size distribution (50–150 nm) and superior preservation of functional markers like CD9 and CD81 (Lu et al., 2021; Ma C.-Y. et al., 2024). Establishing these specific yield and purity metrics is essential for true GMP-compliant manufacturing (Chattopadhyay et al., 2025). For scaffolds, differences in biomaterials (e.g., chitosan, GelMA, collagen), architecture, and mechanical properties alter cell viability and matrix deposition, underscoring the need for standardized fabrication, seeding, and evaluation protocols (Wang N. et al., 2022; Ohnishi et al., 2023). Recent bibliometric analyses highlight growing research but persistent reporting gaps, emphasizing the need for international consensus on reference standards across cells, exosomes, scaffolds, and outcome measures to enable reproducible and clinically translatable therapies (Zhao J. et al., 2025).

### 6.2 Long-term safety considerations

Safety concerns include tumorigenicity, ectopic differentiation, and immune rejection, warranting strict long-term evaluation. Tumorigenicity is a particular risk with immortalized or genetically modified MSCs due to potential chromosomal instability and malignant transformation, highlighting the need for careful genetic monitoring before clinical use (Yamaguchi et al., 2024). MSCs may also undergo ectopic differentiation and form unintended tissues, although this can be reduced by applying controlled differentiation cues within bioengineered constructs (Nakielski et al., 2023; Sun et al., 2023). Immune rejection remains a challenge for allogeneic MSCs because surface antigen disparities may activate host immunity (Choi et al., 2020; Zhou et al., 2023), whereas MSC-derived exosomes may mitigate these risks by lacking MHC molecules and provoking minimal immune activation (Bhujel et al., 2022). To ensure safety, long-term surveillance should incorporate imaging, histology, and molecular assays to detect delayed adverse events (Mesa Bedoya et al., 2024), and standardized manufacturing with structured post-implantation monitoring remains essential for clinical translation (Zhang et al., 2022a).

### 6.3 Regulatory and manufacturing barriers

Good manufacturing practice (GMP) standards for MSC therapies, exosomes, and bioengineered scaffolds are still evolving and remain poorly adapted to these advanced biologics, creating major barriers to translation. Transitioning to GMP-compliant manufacturing presents critical bottlenecks. For exosomes, conventional isolation (e.g., ultracentrifugation) is unscalable and operator-dependent (Ma C.-Y. et al., 2024). To achieve high-yield, standardized production, exploratory solutions increasingly utilize 3D hollow-fiber bioreactors coupled with tangential flow filtration (TFF) (Chua et al., 2022). Similarly, industrial scaffold production is hindered by batch variability and severe biomechanical degradation caused by conventional sterilization (e.g., gamma irradiation) (Zhang J. et al., 2025). To preserve structural integrity during mass production, current strategies implement automated, enclosed 3D bioprinting combined with non-destructive sterilization (e.g., supercritical CO_2_ or cold plasma) (Mohd Isa et al., 2022a; Zhang J. et al., 2025). Resolving these manufacturing bottlenecks and establishing harmonized guidelines remain essential to accelerate clinical approvals (Chattopadhyay et al., 2025). Standardized processes, robust QC systems, and updated regulatory frameworks are essential to enable clinical translation.

### 6.4 Emerging directions in regenerative strategies

Moving beyond the isolated application of individual therapies, the future of intervertebral disc (IVD) repair relies on a synergistic “cell-material-molecule” cross-integration paradigm. For instance, rather than applying cells and scaffolds independently, the combination of 3D bioprinting with gene-edited MSCs (e.g., engineered to overexpress anti-inflammatory genes) enables the fabrication of customized composite discs that mimic native architecture while providing a hyper-active biological core (Binch et al., 2021; Zhu et al., 2021; Kim et al., 2023). Moving forward, the “cell-material-molecule” paradigm must be anchored in precise mechanistic loops where smart biomaterials provide matched responses to specific microenvironmental stressors. Specifically, for the acidic niche (pH < 6.8), hydrogels incorporating acid-labile linkages (e.g., hydrazone or acetal bonds) are engineered to undergo rapid hydrolysis or protonation, triggering network swelling and the on-demand release of therapeutic payloads such as anti-inflammatory exosomes (Ma T. et al., 2024; Zhang J. et al., 2025). To counteract severe oxidative stress, biomaterials utilizing ROS-cleavable linkages (e.g., thioketal or boronate ester bonds) can simultaneously scavenge pathological ROS and trigger the release of anabolic molecules like TGF-β (Tang et al., 2025). Furthermore, to address mechanical overload, the implementation of energy-dissipating hydrogels featuring dynamic reversible crosslinking (e.g., Schiff base bonds or hydrogen bonding) allows the scaffold to periodically break and reform under high physiological loads. This dynamic stress relaxation effectively shields the encapsulated MSCs from mechanical-induced apoptosis, ensuring sustained cell viability within the biomechanically demanding IVD environment (Mordechai et al., 2023; Nakielski et al., 2023).

Furthermore, future research within this integrated framework must pivot towards explicitly defined core targets. At the genetic-epigenetic level, deciphering the regulatory networks of key exosomal miRNAs/lncRNAs is paramount for directing matrix synthesis (Li W. et al., 2022; Xu G. et al., 2023). Concurrently, at the pathway level, synergistically targeting the NLRP3 inflammasome to dismantle cytokine cascades (Zhang et al., 2020; Zhao et al., 2021), alongside leveraging the HIF-1α signaling pathway to boost hypoxic adaptability (He et al., 2021), represent the most critical technical combinations for follow-up research. Ultimately, integrating materials science, gene editing, and artificial intelligence into this unified roadmap will drive the next-generation of precise and functional clinical IVD therapies (Hong, 2022; Trapana et al., 2024).

### 6.5 Future vision

**FIGURE 4:**
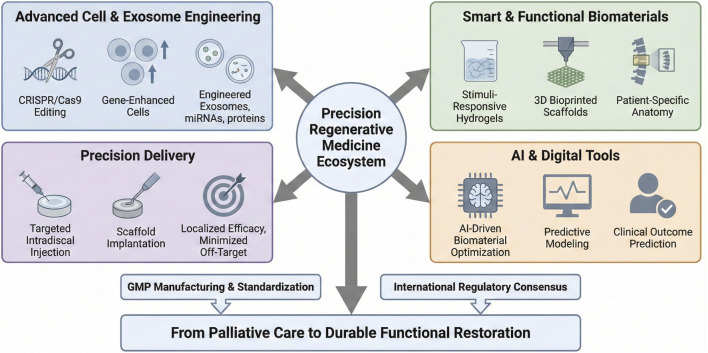
Integrated Framework for Precision Regenerative Medicine in IVDD. This schematic outlines the convergence of multidisciplinary strategies aimed at transitioning from palliative care to functional disc restoration. The framework is supported by four core pillars: (1) Advanced Cell and Exosome Engineering, utilizing gene editing (e.g., CRISPR/Cas9) to enhance the survival and therapeutic potency of mesenchymal stem cells (MSCs) and their secreted vesicles; (2) Functional Biomaterials, employing smart, stimuli-responsive hydrogels and 3D bioprinted scaffolds to provide mechanical support and a pro-regenerative niche; (3) Precision Delivery, optimizing intradiscal injection and scaffold implantation to ensure targeted therapeutic action; and (4) AI-Driven Optimization, leveraging artificial intelligence to refine biomaterial-cell interactions and predict clinical outcomes. By integrating these pillars, this precision medicine approach aims to overcome current translational barriers and achieve durable, patient-specific regeneration of the degenerative disc microenvironment.

Advances in cell engineering, smart biomaterials, precision delivery, and artificial intelligence (AI)-driven optimization are converging toward a unified new paradigm of precision regenerative medicine for IVDD (Figure 4). To overcome the inherent challenges of poor cell survival in the hostile disc niche, the future relies on an integrated ecosystem combining genetically enhanced cells, engineered exosomes, and supportive biomaterial scaffolds (Hong, 2022; Trapana et al., 2024). By refining precision delivery techniques—such as minimally invasive intradiscal injection and targeted scaffold implantation—this multidisciplinary convergence promises to maximize therapeutic efficacy and successfully transition clinical practice from palliative symptom management to durable, functional disc restoration (Cheng et al., 2018; Nakielski et al., 2023).

## 7 Conclusion

While isolated therapies face substantial translational barriers, their strategic “cell-material-molecule” convergence represents a transformative regenerative solution for IVDD. A critical synthesis of current evidence reveals an unresolved controversy between immunomodulatory symptom relief and true structural regeneration, necessitating a paradigm shift across three clinical dimensions:

Diagnostic implications: Precise staging of the degenerative microenvironment (e.g., assessing hypoxia and acidity severity) is essential to tailor patient-specific biomaterial and exosome therapies.

Therapeutic implications: Overcoming the gap between *in vitro* anabolism and *in vivo* biomechanical demands mandates the integration of load-bearing, biologically responsive composite scaffolds.

Prognostic implications: Transitioning from palliative pain management to durable functional restoration ultimately relies on overcoming GMP manufacturing hurdles and standardizing safety protocols.
